# Local immune responses to tuberculin skin challenge in *Mycobacterium bovis* BCG-vaccinated baboons: a pilot study of younger and older animals

**DOI:** 10.1186/s12979-021-00229-w

**Published:** 2021-04-07

**Authors:** Julia M. Scordo, Tucker J. Piergallini, Nicole Reuter, Colwyn A. Headley, Vida L. Hodara, Olga Gonzalez, Luis D. Giavedoni, James F. Papin, Joanne Turner

**Affiliations:** 1 Texas Biomedical Research Institute, San Antonio, TX USA; 2 The University of Texas Health Science Center of San Antonio, San Antonio, TX USA; 3 Biomedical Sciences Graduate Program, The Ohio State University, Columbus, OH USA; 4 University of Oklahoma Health Sciences Center, Oklahoma City, OK USA

**Keywords:** Aging, Vaccine immunity, Tissue recall responses, Tuberculin skin test

## Abstract

Individuals over the age of 65 are highly susceptible to infectious diseases, which account for one-third of deaths in this age group. Vaccines are a primary tool to combat infection, yet they are less effective in the elderly population. While many groups have aimed to address this problem by studying vaccine-induced peripheral blood responses in the elderly, work from our lab and others demonstrate that immune responses to vaccination and infectious challenge may differ between tissue sites and the periphery. In this pilot study, we established an in vivo delayed-type hypersensitivity model of *Mycobacterium bovis* BCG vaccination and tuberculin skin test in two adult and two aged baboons. Vaccination generates BCG-specific immune cells that are recruited to the skin upon tuberculin challenge. We tested short term recall responses (8 weeks post-vaccination) and long term recall responses (25 weeks post-vaccination) by performing skin punch biopsies around the site of tuberculin injection. In short term recall responses, we found increased oxidation and decreased production of immune proteins in aged baboon skin at the site of TST challenge, in comparison to adult skin. Differences between adult and aged animals normalized in the long term response to tuberculin. In vitro, aged peripheral blood mononuclear cells had increased migration and functional responses to antigen-specific stimulation, suggesting that age-related changes in the tissue in vivo impairs aged immune recall responses to antigenic challenge. These findings highlight the impact of age-associated changes in the local tissue environment in memory recall responses, which may be more broadly applied to the study of other tissues. Moreover, these findings should be considered in future studies aimed at understanding and improving aging immune responses to vaccination and tissue challenge.

**Supplementary Information:**

The online version contains supplementary material available at 10.1186/s12979-021-00229-w.

## Background

By 2060, individuals aged 65 and older will constitute approximately one-fourth of the U.S. population [1]. This shift in demographics will have significant financial and public health consequences. As we age, so do many aspects of our immune system resulting in decreased vaccine protective efficacy and increased susceptibility to infection [2, 3]. This has been recently highlighted by the increased susceptibility of the elderly to SARS-CoV-2, in addition to other infections, such as influenza and bacterial pneumonia [4, 5]. To improve health outcomes in our aging population, we must gain a better understanding of age-associated changes in immune cell and tissue function in the elderly. With increasing age immune cells in the periphery shift to more differentiated CD4 and CD8 T cell subsets with altered function and acquisition of senescence markers [6–9]. Additionally, increased systemic levels of inflammation with advanced age contribute to age-related diseases [8, 10]. While peripheral blood studies have led to a greater understanding of the immunological aging process, much less is known about the impact of age on memory responses in tissue compartments. Studies, including those from our lab, have shown that immune responses in the periphery and at the site of infection can differ [11–13].

The skin is a site of frequent exogenous challenge and contains resident and recruited cells of both the innate and adaptive arms of the immune system [14]. Age-associated changes in the skin tissue, i.e. increased inflammatory mediators and reactive oxygen species (ROS), decreased chemokine production by resident cells, and altered skin vasculature, reduces cutaneous immunity and increases risk of skin infections [14–17]. In humans, studies have shown that development of delayed-type hypersensitivity responses to antigen challenge, including TST, are reduced and/or delayed in the elderly due in large part to high levels of inflammation that blunt recall memory responses in the skin [18–20]. The skin is therefore an ideal model to study age-related changes in tissue immunity. Because elderly tissue samples in humans are difficult to obtain and limited, it is necessary to develop novel models to study aging immune responses in the tissue.

Here we took advantage of the high homology between the baboon and human immune system to establish an in vivo tissue model to study immune responses to vaccination [21, 22]. A group of two adult and two aged baboons were vaccinated with *Mycobacterium bovis* BCG (BCG) and the classical delayed-type hypersensitivity (DTH) reaction of the tuberculin skin test (TST) was utilized to evaluate antigen-specific recall responses to the skin in timed biopsies [23]. We tested short term (ST) recall responses (8 weeks post-vaccination) as well as long term (LT) recall responses (25 weeks post-vaccination) to evaluate the impact of age on vaccine-induced tissue immunity.

## Results and discussion

### Establishing an in vivo model of tuberculin recall response in BCG-vaccinated baboons

**Fig. 1:**
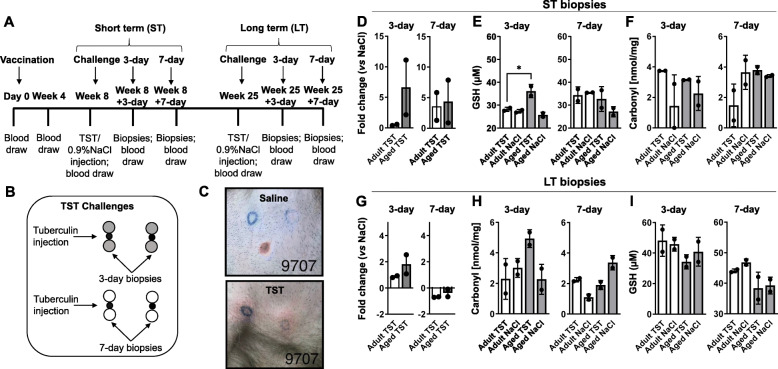
An in vivo model of tuberculin recall response in *Mycobacterium bovis* BCG-vaccinated baboons. **a** Study timeline: 2 adult and 2 aged baboons were BCG-vaccinated and challenged with tuberculin (TST) or saline (NaCl) for determination of the short term (ST) recall response. Skin biopsies were performed 3 days and 7 days post-challenge. Long term (LT) responses were tested by challenge and biopsies performed at week 25, week 25 + 3 days, and week 25 + 7 days, as indicated. Peripheral blood was collected at the times indicated for PBMC isolation. **b** Schematic of TST challenges and biopsy sites on the chest of vaccinated baboons. Saline injection sites served as the control for antigen-specific responses. **c** Representative images of saline (left) and TST (right) injection sites, indicated by blue circle, on the chest of vaccinated baboons, showing positive responses to TST. **d** Levels of superoxide detected by EPR in adult and aged baboon skin tissue. Shown is the fold change of superoxide in TST biopsies (TST) vs saline (NaCl) biopsies from ST 3-day (left) and 7-day (right) biopsies. **e** Reduced glutathione (GSH) in ST 3-day (left) and 7-day (right) biopsies, determined by ELISA. **f** Protein carbonyl levels in ST 3-day (left) and 7-day (right) biopsies, determined by ELISA. **g** Superoxide in baboon skin tissue in response to TST (fold change vs saline) from LT 3-day (left) and 7-day (right) biopsies. **h** Protein carbonyl levels in LT 3-day (left) and 7-day (right) biopsies. **i** GSH in LT 3-day (left) and 7-day (right) biopsies. One-way ANOVA post-Tukey analyses Adult TST vs Aged TST, *p < 0.05

To evaluate the impact of age on vaccine-induced recall immunity to the skin, we vaccinated 4 baboons (two adult and two aged, according to age ranges previously described [24], (Table 1) with BCG via the intradermal route at a dose of 5-8 × 10^5^ CFUs (Fig. 1a; See Supplementary Fig. 1A, Additional File 1).

**Table 1:** Table of sex, date of birth, and age of animals in the study

Animal #	Sex	Date of Birth	Age (Years)
1	Male	11/02/2007	11.47
2	Male	12/18/2007	11.34
3	Male	02/01/2003	16.22
4	Male	02/13/2004	15.19

For adult and aged baboons in the study, sex, date of birth, and age in years is provided

We tested ST recall responses to BCG with TST, or 0.9% saline skin injection (control), at 8 weeks post-vaccination (Fig. 1a) by performing two TST and two saline skin injections on the chest of each animal (Fig. 1b). This time period of 8 weeks allows for development of immune memory to BCG [25]. Immune recall responses were determined by performing skin punch biopsies surrounding the site of TST or saline injection (Fig. 1b, c) at two time points post-TST (ST 3-day biopsy, and ST 7-day biopsy) (Fig. 1a). We chose these two time points post-TST for biopsy collection to account for age-related differences in kinetic recall response to TST. In support of this, clinical responses to TST manifest within 3 days of TST; however, maximal cellular infiltration doesn’t occur until 7 days [23]. Moreover, TST responses in the elderly are commonly delayed or reduced [18, 26]. A second challenge was performed 25 weeks after BCG-vaccination to determine LT recall immune responses (Fig. 1a), with two TST and two saline skin injections performed on a different region of the chest. For determination of LT recall responses, LT 3-day biopsies and LT 7-day biopsies post-TST were also obtained (Fig. 1a). Saline injection did not induce any changes in skin biopsies obtained from adult and aged BCG-vaccinated baboons at any time point ST or LT (included in figures as a baseline measure; shown in Fig. 1c, *left*), supporting that the injection procedure did not induce non-specific responses. Additionally, animal weight in both age groups was unchanged throughout the duration of the study (See Supplementary Fig. 1B, Additional File 1).

### Increased oxidation and altered immune mediator production in skin from aged baboons, in response to TST

Work from our labs has shown that the elderly lung environment is pro-oxidative and inflammatory, leading to increased susceptibility to *Mycobacterium tuberculosis* (*M.tb*) infection [27–29]. Moreover, cutaneous infections are more commonly observed in older individuals due to age-related changes in the skin, such as decreases in structural integrity and cellularity and increases in ROS [14, 19, 30, 31]. A predominant source of ROS in the skin is superoxide anion [15]. We evaluated superoxide levels in the skin of BCG-vaccinated adult and aged baboons in response to TST using electron parametric resonance (EPR). In the ST response, we observed increased superoxide in aged skin from 3-day biopsies (Fig. 1d, *left*). Oxidation in adult skin increased in the ST 7-day biopsy time point reaching levels comparable to that of aged skin (Fig. 1d, *right*). In the skin, glutathione is one of the most important antioxidants capable of reducing ROS levels [16]. Higher levels of reduced glutathione were found in aged skin from ST 3-day biopsies in response to TST (Fig. 1e, *left*). Similar to oxidation levels, reduced glutathione were higher in adult skin from ST 7-day biopsies, reaching the levels of those observed in the aged skin (Fig. 1e, *right*). Lastly, we measured protein carbonyls in the skin, which reflect the degree of tissue oxidative damage [16]. We saw no differences in protein carbonyls present in the skin between our age groups in ST biopsies (Fig. 1f). Skin obtained from the LT challenge time point showed a trend increase in superoxide levels and protein carbonyls in aged skin from LT 3-day biopsies (Fig. 1g & h, *3-day*), with oxidation levels decreasing in both age groups in LT 7-day biopsies (Fig. 1g & h, *7-day*). Reduced glutathione levels showed a trend increase in adult skin in response to TST in LT 3-day and 7-day biopsies post-TST (Fig. 1I). These results suggests that older animals mount an early, enhanced oxidative response not observed in adult animals.

**Fig. 2:**
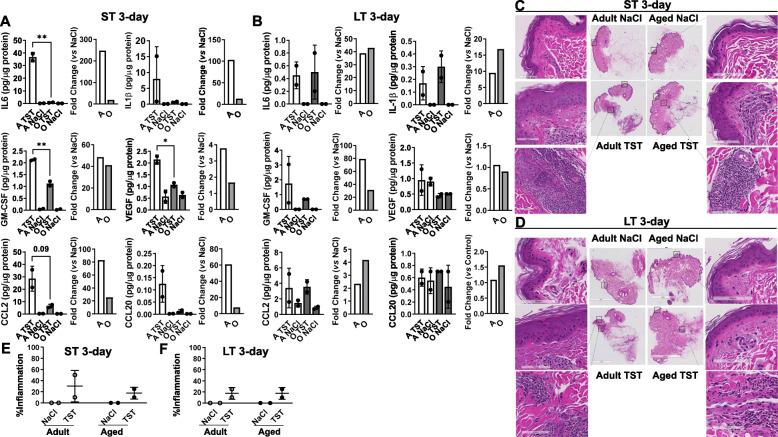
Decreased early immune mediator production and less inflammation in aged baboon skin challenged with TST. Levels of immune proteins in (**a**) ST 3-day biopsies and (**b**) LT 3-day biopsies from adult (A) and aged (O) vaccinated baboons were detected by Luminex assay. Shown for each analyte: protein concentration normalized per μg of protein in skin tissue homogenates (left) and the fold change of TST protein levels vs saline (right). One-way ANOVA post-Tukey analyses Adult TST vs Aged TST, *p < 0.05, **p < 0.01. H&E stained skin tissue from adult and aged baboons was evaluated for inflammation, cell infiltration, and skin structural changes in ST 3-day biopsies (**c**) and LT 3-day biopsies (**d**). Representative images are shown of adult (left) and aged (right) skin tissue in response to NaCl (top) and TST (bottom) for ST 3-day biopsies (**c**) and LT 3-day biopsies (**d**). Percent affected inflammation is quantified in ST 3-day biopsies (**e**) and LT 3-day biopsies (**f**)

Elderly skin has been shown to have higher basal levels of inflammatory cytokines [19]. When challenged, early and non-specific inflammatory responses in the skin have been observed [32], blunting subsequent antigen-specific responses. We next evaluated cytokine, chemokine, and growth factors present in aged and adult skin from BCG-vaccinated baboons. In response to TST, we observed a significant decreased production of cytokines, chemokines, and growth factors in the skin of aged baboons in the ST 3-day biopsies (Fig. 2a). In both age groups, immune protein levels were decreased in ST 7-day biopsies (See Supplementary Fig. 2A, Additional File 1). During the LT recall response, TST-challenged 3-day biopsies from aged skin responded similarly to TST-challenged adult 3-day biopsies, although aged skin had higher levels of IL1β and a fold change increase in several chemokines (Fig. 2b). Overall, the relative magnitude of immune protein levels in LT biopsies was less than levels of immune proteins observed in the ST response to TST, suggesting that recall responses for both age groups decreased over time (Fig. 2b; See Supplementary Fig. 2B, Additional File 1).

### Cell infiltration and skin histological analysis is similar between aged and adult skin

Histological analyses of skin using hematoxylin and eosin staining methods from BCG-vaccinated aged and adult animals was performed to determine the extent of cellular infiltration. Inflammation was visually assessed and quantified as percentage inflammation by a board-certified pathologist. In the ST recall response, TST-challenged adult skin from 3-day biopsies had more percent affected inflammation in comparison to aged animals supporting the elevated cytokines detected at this same time point (Fig. 2c,e). Inflammation decreased to comparable levels in ST 7-day biopsies in response to TST (See Supplementary Fig. 3, Additional File 1). We observed equal levels of inflammation in TST-challenged skin from the LT 3-day and 7-day biopsies in our age groups (Fig. 2d,f; See Supplementary Fig. 4, Additional File 1). Skin from aging individuals has known structural changes including decreased extracellular matrix components and reduced epidermal thickness [19, 33], so we also evaluated structural alterations. Adult ST 3-day skin biopsies showed minimal increase in epidermal thickness by visual analysis, which is supported by the slight increase in inflammation in adult skin in response to TST, relative to aged skin (Fig. 2c,e).

### Aged PBMCs have increased migration in response to skin tissue homogenates

**Fig. 3:**
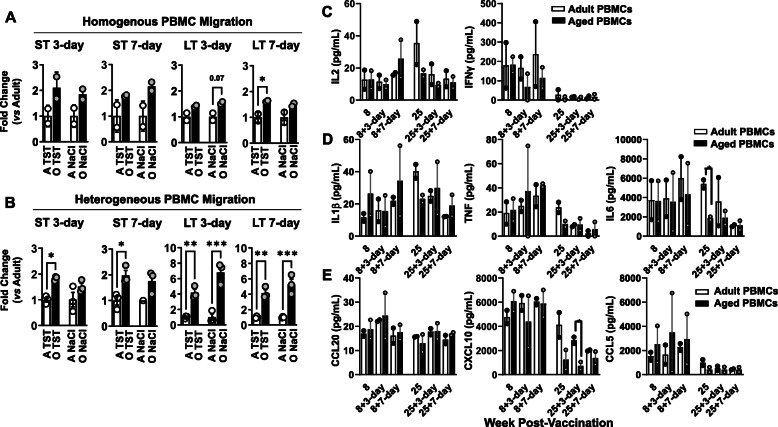
Increased aged PBMC migration in response to skin homogenates and functional PBMC responses to antigen. **a** Homogeneous migration of adult and aged PBMCs in response to skin tissue homogenates from TST and NaCl ST 3-day and 7-day biopsies and LT 3-day and 7-day biopsies. A is adult; O is aged. Each dot represents one animal. **b** Heterogeneous migration of adult and aged PBMCs in response to skin tissue homogenates from TST and NaCl ST 3-day and 7-day biopsies and LT 3-day and 7-day biopsies. Each dot represents a replicate, performed in triplicate, from adult or aged PBMCs by age group. Shown is fold change vs adult for homogeneous (A) or heterogenous (B) experimental setup. Student’s *t* test Adult TST vs Aged TST and Adult NaCl vs Aged NaCl, *p < 0.05, **p < 0.01, ***p < 0.001. **c**-**e** PBMCs from adult and aged vaccinated baboons from the time points indicated were stimulated with CFP for 5 days. Supernatants were collected and antigen-specific responses were detected by Luminex for production of (**c**,**d**) cytokines and (**e**) chemokines. Student’s *t* test Adult vs Aged PBMCs, *p < 0.05

To test if advanced age impacts recall responses through changes in cell migration, we tested peripheral blood mononuclear cell (PBMC) chemotaxis in response to mediators present in skin tissue. Adult or aged PBMC migration was evaluated in response to adult skin or aged skin homogenates, respectively, termed homogenous chemotaxis (Fig. 3a). Moreover, heterogeneous chemotaxis was evaluated by testing adult PBMC migration to aged skin homogenates and aged PBMC migration to adult skin homogenates (Fig. 3b). Data are shown as fold-increase in the response of PBMCs from aged baboons relative to adult. In response to TST skin tissue, aged PBMCs had increased migration in both homogenous and heterogeneous migration assays, relative to adult PBMCs (Fig. 3a & b, *Adult TST* vs *Aged TST*). This increase of aged PBMC migration was observed regardless of biopsy time point (ST vs LT 3-day and 7-day biopsies). To account for non-specific migration in response to immune mediators present in resting skin, homogenous and heterogeneous cell migration was also tested in response to 3-day and 7-day biopsies from saline injected skin (Fig. 3a&b, *Adult NaCl & Aged NaCl*). In response to saline skin tissue, aged PBMCs had higher levels of cell migration than adult PBMCs. These findings suggest that in vitro aged BCG-vaccinated PBMCs have better capacity to migrate in response to TST biopsy homogenates. Also, these findings suggest that the PBMC response was increased independent of the tissue-specific milieu (TST vs saline). Based on these findings, we believe that age-associated changes in the tissue structure in vivo impacts cell migration that is not detected using an in vitro cell assay that requires tissue disruption. It is important to note that these migration studies were performed using PBMCs, a mixture of cell types, as the input cell source, and future phenotypic analysis of migrated cells may shed light on aged-related differences in migrating cell populations. While it is possible that a yet unidentified chemoattractant is driving the enhanced migration we observed in aged PBMCs, this is a less likely explanation because the chemoattractant would need to be found in both adult and aged skin, and at both basal levels and in response to TST.

### PBMC from aged baboons have functional responses to antigen stimulation

Following our observations of increased migration of PBMC from aged baboons, we tested functional responses of PBMCs from our vaccinated animals in response to in vitro stimulation with mycobacterial antigen. In the ST (Fig. 3c-e, *Weeks 8, 8 + 3-day, 8 + 7-day*) and LT (Fig. 3c-e, *Weeks 25, 25 + 3-day, 25 + 7-day*) response to TST, no differences in antigen-specific T cell cytokines (IL2 and IFN-γ) were observed between age groups (Fig. 3c). Adult PBMCs had transient increased production of the pro-inflammatory cytokines IL1β, TNF and IL6, although this was from blood collected prior to LT skin challenge and therefore not induced by TST (Fig. 3d). Adult PBMCs from the LT TST response had increased CXCL10, although this was only observed at one time-point and not sustained throughout the LT response (Fig. 3e). At all time points tested, aged and adult PBMC baseline responses (media alone) were below the limit of detection.

Due to the nature of a pilot study, this work is limited by a small sample size of four total animals and a modest age group difference between our adult and aged animals. In our study, adult animals were defined as less than 12 years old; however, other aging baboon studies define adult animals as 5 years and older [34]. Despite these limitations we observed robust tissue changes between our age groups and established the use of this model for future studies in a larger group of animals. Our findings demonstrate that age-related changes in the skin tissue (increased oxidation, decreased immune proteins, and decreased inflammation) result in reduced early immune responses to antigenic challenge at the tissue site. This suggests that studying the impact of increased age on tissue immune responses in response to vaccination or infection may be a more successful approach to understand immunity in the elderly.

## Methods

### Animal procedures

Studies were conducted in four (two adult and two aged) baboons from the conventional colony at The University of Oklahoma Health Sciences Center (OUHSC) [Fig. 1]. All animals were housed in Animal Biosafety Level 1 (ABSL1) facilities at OUHSC for the duration of the study. All procedures were approved by the University Institutional Animal Care and Use Committee at OUHSC and the OUHSC Biosafety Committee. Animals were vaccinated with *Mycobacterium bovis* BCG (Pasteur strain, ATCC, Manassas, VA) via the intradermal route in the upper arm at a dose of 5 × 10^5^ CFUs. Skin tests were performed on animals at the times indicated in Fig. 1 by injecting 100 μL of tuberculin (Colorado Serum Company, Denver, CO) containing 5 tuberculin units of purified protein derivative (PPD). Saline injections (100 μL of 0.9% NaCl, Baxter International Inc., Deerfield, IL) were performed in animals to serve as negative controls. All skin tests were performed in the chest skin of animals according to the diagram in Fig. 1. All animals had positive TST responses in the study as determined by visual observation (Fig. 1c). At 72 h and 7-days post-skin test challenge, 8 mm skin punch biopsies (ThermoFisher Scientific, Waltham, MA) were obtained from the sites of tuberculin and saline injection (schematic in Fig. 1). All biopsies were shipped overnight to Texas Biomedical Research Institute (Texas Biomed) for downstream processing. Blood was collected in sodium heparin-vacutainers according to the timeline in Fig. 1 and shipped overnight to Texas Biomed for processing. All procedures were performed under anesthesia (10 mg/kg Ketamine and 0.05–0.5 mg/kg Acepromazine, Covetrus, Portland, ME), and included monitoring of weight, body temperature, heart rate, respiration rate and capillary refill time.

### Preparation and storage of skin punch biopsies

Immediately following collection at OUHSC, each 8 mm skin biopsy was cut in half using a pathology blade and biopsy halves were stored in 10% neutral buffered formalin (ThermoFisher Scientific), snap-frozen in liquid nitrogen, or prepared for electron paramagnetic resonance (EPR) analysis. Remaining skin tissue was banked for future analysis. For EPR analysis, skin biopsies were further divided into two pieces, weighed, and incubated with the following: 1) 400 μM CMH-hydrochloride (Enzo Life Sciences, Farmingdale, NY) or 2) 400 μM CMH + 500 nM rotenone (MP Biomedicals, Santa Ana, CA) + 100 μM antimycin A from Streptomyces sp. (MilliporeSigma, Burlington, MA) (CMH + RA) for 30 min at 37 °C. After 30-min incubation, CMH or CMH + RA solution was removed from the tissue and stored in a cryovial. Both tissue and CMH solutions were snap-frozen in liquid nitrogen. Biopsies were shipped on dry ice (snap-frozen tissues) or at room temperature (tissues in formalin) to Texas Biomed for downstream processing.

### Preparation of tissue homogenates from skin biopsies

Skin punch biopsies (snap-frozen in liquid nitrogen) were homogenized in Lysing Matrix D tubes (MP Biomedicals) in Tissue Extraction Reagent I (ThermoFisher Scientific) with cOmplete Mini Protease Inhibitor Cocktail (MilliporeSigma). Protein content was determined by Pierce BCA protein assay kit (ThermoFisher Scientific), according to kit instructions.

### Luminex assay measurement of immune mediators

Immune mediators were measured in cell supernatants, plasma, and tissue homogenates using the following Luminex assays cross-reactive with baboons: NHP XL Cytokine for detection of CCL5, CCL20, CXCL10, IL1β, IL2, IL6, TNFα, and IL8, and Human XL Cytokine for detection of CCL2, CXCL10, IL8, IL1β, and IL6 (R&D Systems, Minneapolis, MN), according to kit instructions. For detection of IFN-γ and IL2, monkey interferon-gamma (IFN-γ) ELISA kit and monkey IL-2 ELISA kit (Mabtech, Inc., Cincinnati, OH) were performed, according to kit instructions. For tissue homogenates, analyte levels were normalized to protein content in homogenates (pg analyte per μg protein), as determined by BCA assay.

### Measurement of tissue oxidation levels

For superoxide determination by EPR, CMH and CMH + RA tissue supernatants were thawed and loaded into a quartz cell. Superoxide levels in the samples were determined by EPR. EPR spectra were obtained on the Bruker EMXnano ESR system (Bruker Corporation, MA, USA) using the following parameters: Frequency, 9.636541 GHz; Center Field, 3435.30 G; Modulation Amplitude, 2.000 G; Power 0.3162 mW; Conversion Time of 40.00 ms; Time Constant of 1.28 ms; Sweep Width of 100.0 G; Receiver Gain at 40 dB; and 1 total number of scans. Samples were baseline corrected relative to CMH only (control). Area under the curve for each sample was then determined.

Protein carbonyls in skin tissue homogenates were determined using a Oxiselect Protein Carbonyl ELISA kit (Cell Biolabs, Inc., San Diego, CA), according to kit instructions. Carbonyl levels were normalized to protein content in homogenates, as determined by BCA assay.

Reduced glutathione (GSH) was determined in skin tissue homogenates using a GSH/GSSG Ratio Detection Assay kit (Abcam, Cambridge, MA), per kit instructions. Glutathione levels were normalized to protein content in homogenates, as determined by BCA assay.

### Isolation of PBMCs from whole blood

Blood was diluted with 1X PBS and Lymphocyte Separation Media (Corning Life Sciences, Tewksbury, MA) was slowly dispensed underneath the blood, followed by centrifugation at 950 x g for 20 min at room temperature with disconnected brake. After centrifugation, the interface containing PBMCs was transferred to a new tube, washed once with 1X PBS, and red blood cells were lysed (freshly prepared lysis solution containing 0.15 M NH_4_Cl, 10 mM KHCO_3_, 0.1 mM Na_2_-EDTA). Cells were washed twice to remove lysis solution and re-suspended in complete medium: 1X RPMI 1640 supplemented with 25 mM HEPES (MilliporeSigma), 10% heat-inactivated fetal bovine serum (Atlas Biologicals, Fort Collins, CO), 1% HyClone, 1% L-glutamine, and 1% MEM Non-Essential Amino Acids (all from ThermoFisher Scientific).

### PBMC stimulation

For cell stimulations, PBMCs were plated at a final concentration of 250,000 c/well. Cells were stimulated for up to 5 days in the presence of media or *Mycobacterium tuberculosis* culture filtrate protein (CFP), which contains BCG cross-reactive antigens (BEI Resources, Manassas, VA). For 24–28 h incubations, CFP was used at a concentration of 20 μg/mL. For 5 day incubations, CFP was used at a concentration of 10 μg/mL. Supernatants were collected at the end of the incubation and stored at − 80 °C.

### Freezing and thawing of PBMCs

To prepare PBMCs for freezing, cells were re-suspended at a concentration of 10 × 10^6^ c/mL in freezing medium: 85% heat-inactivated FBS + 10% DMSO + 5% of glucose 45% solution (MilliporeSigma). Cryovials containing 1 mL cell suspension were transferred to a pre-chilled Mr. Frosty and stored at − 80 °C for no longer than 48 h. Vials were then transferred to liquid nitrogen for long term storage. To thaw PBMCs, pre-warmed complete medium was supplemented with 0.2 μl/ml of Benzonase HC (MilliporeSigma). Cryovials containing frozen PBMCs were quickly thawed in a 37 °C water bath. After thaw, 1 mL cell medium + 0.2 μl/ml Benzonase was added to each cryovial and the volume was transferred to a 15 mL conical tube. Cells were centrifuged at 250 x g for 7 min at room temperature. Cells were then re-suspended in complete cell medium and counted for downstream analyses.

### PBMC migration to skin tissue homogenates

PBMC migration was determined using the CytoSelect 96-Well Cell Migration Assay (5 μM, Fluorometric Format [Cell Biolabs, Inc]), according to manufacturer’s instructions. Briefly, skin tissue homogenates with known protein content according to BCA assay were prepared at 50 μg protein in a final volume of 150 μL serum-free medium (1X RPMI 1640 supplemented with 25 mM HEPES, 1% HyClone, 1% L-glutamine, and 1% MEM Non-Essential Amino Acids). Homogenates were added to the bottom chamber of the cell migration plate according to homogenous (Adult to Adult; Aged to Aged) or heterogeneous (Adult to Aged; Aged to Adult) experimental design. Then 500,000 PBMCs in serum-free media were added to the top chamber of the cell migration plate and incubated for 6 h at 37 °C. At the end of the incubation period, cell detachment solution was added to the cell harvesting plate. Next, media in the top chamber wells from cell migration plate containing non-migrating cells was discarded and the cell migration top chamber plate was inserted into the cell harvesting tray for 30 min at 37 °C to detach cells. The bottom chambers of the cell migration plate were set aside. After the cell detachment incubation, 75 μL of detachment media (from harvesting plate) and 75 μL of media (from bottom chambers of the cell migration plate) was added to a clear 96-well plate. Lysis buffer and dye solution (from kit) was added to each well of the clear 96-well plate and incubated for 20 min at room temperature. After the incubation, 150 μL from the clear 96-well plate was moved to a clear bottom, black plate and fluorescent was read in a plate reader with a 485 nm/ 538 nm filter and 530 nm cutoff.

### Histological analysis of skin tissue

Skin biopsies fixed in 10% neutral buffered formalin were paraffin-embedded, sectioned at 4 μm thickness, stained with hematoxylin and eosin using standard methods, and evaluated by a board-certified veterinary pathologist at Texas Biomed. For percent affected inflammation, the following scores were used for grading: 1 = < 10%, 2 = < 25%, 3 = < 50%, 4 = < 75%, 5 = > 75%. Paraffin-embedded skin biopsies were also processed for trichrome elastin staining per standard methods, and similarly evaluated. Tissue images were viewed using Image Scope × 64 software.

### Statistical analyses

Data analysis, graphing, and statistical analysis was performed using GraphPad Prism version 7–9 (La Jolla, CA). For statistical analysis the following were used as described in the figure legends: One-way ANOVA and Tukey’s post hoc correction for multiple-testing and Student’s *t* test for testing of means between two groups. Statistical differences between groups were reported significant when the *p*-value is less than or equal to 0.05. The data are presented in mean ± SEM for *n* = 2 animals per age group.

## Supplementary Information

**Additional file 1:.** SCORDO-TURNER-03192021.docx. Additional File 1 contains supplementary figures #1–4 to the body of the manuscript.

## Data Availability

All data generated or analyzed during this study are included in this published article (and its supplementary information files).

## Abbreviations

BCG: *Mycobacterium bovis* BCG
TST: Tuberculin skin test
DTH: Delayed-type hypersensitivity
ST: Short term
LT: Long term
ROS: Reactive oxygen species
PBMC: Peripheral blood mononuclear cells
