# Supplementary material for: Local immune responses to tuberculin skin challenge in Mycobacterium bovis BCG-vaccinated baboons: a pilot study of younger and older animals
Source: Immun Ageing. 2021 Apr 7;18:16. doi: 10.1186/s12979-021-00229-w (PMC8024439; doi:10.1186/s12979-021-00229-w)
Supplement: Supplementary file 1 — Additional file 1:. SCORDO-TURNER-03192021.docx. Additional File 1 contains supplementary figures #1–4 to the body of the manuscript. [file 12979_2021_229_MOESM1_ESM.docx]

**Supplementary Figure 1. *Mycobacterium bovis* BCG vaccination dose and animal weight change during study.**

**BCG Inoculum**

**CFUs**

**Weight Fold Change**

**B**

**A**

(**A**) *Mycobacterium bovis* BCG inoculum, determined by colony forming units (CFUs). CFUs were performed by serial dilution followed by incubation on 7H11 agar for 14-21 days. (**B**) Fold change of the weight of adult and aged vaccinated baboons, comparing pre-vaccination and post-vaccination weight.

**Supplementary Figure 2. Levels of immune proteins in adult and aged baboon skin in response to TST.**

****Protein levels from ST 7-day biopsies (**A**) and LT 7-day biopsies (**B**). Each immune mediator is shown as protein concentration normalized per µg of protein in skin tissue homogenates (left) and the fold change of TST protein levels *vs* saline (right). In B, IL6 and GM-CSF were below the level of detection (not detected, N.D.).

**Supplementary Figure 3.** **Skin histological analyses of adult and aged baboon skin in response to ST challenge.**

H&E stained skin tissue from adult and aged baboons was evaluated for percent inflammation in ST 7-day biopsies. Percent affected inflammation is quantified on the left. Representative images (right) are shown of adult (left) and aged (right) skin tissue in response to NaCl (top) and TST (bottom).

**Supplementary Figure 4.** **Skin histological analyses of adult and aged baboon skin in response to LT challenge.**

H&E stained skin tissue from adult and aged baboons was evaluated for percent inflammation in LT 7-day biopsies. Percent affected inflammation is quantified on the left. Representative images (right) are shown of adult (left) and aged (right) skin tissue in response to NaCl (top) and TST (bottom).
